# Sleep SAAF responsive parenting intervention improves mothers’ feeding practices: a randomized controlled trial among African American mother-infant dyads

**DOI:** 10.1186/s12966-022-01366-1

**Published:** 2022-10-01

**Authors:** Erika Hernandez, Justin A. Lavner, Amy M. Moore, Brian K. Stansfield, Steven R. H. Beach, Jessica J. Smith, Jennifer S. Savage

**Affiliations:** 1 Center for Childhood Obesity Research, The Pennsylvania State University, University Park, State College, PA USA; 2 Department of Psychology, Psychology Building, University of Georgia, Athens, GA 30602-3013 USA; 3 Department of Pediatrics, Medical College of Georgia, Augusta, GA USA; 4 Center for Family Research, University of Georgia, Athens, GA USA

**Keywords:** Responsive parenting, Prevention, African American, Infancy, Feeding practices, Childhood obesity

## Abstract

**Background/Objective:**

Parents shape children’s early experiences with food, influencing what is served, children’s food choices, and how much children eat. Responsive parenting (RP) interventions such as INSIGHT have improved maternal infant feeding practices, but have only been tested among predominantly White families. This secondary analysis of data from the Sleep SAAF (Strong African American Families) RCT tests the effects of an RP intervention designed to prevent rapid infant weight gain on African American mothers’ infant feeding practices.

**Methods:**

Primiparous African American mother-infant dyads (*n* = 194) were randomized to an RP or safety control intervention delivered by community research associates at infant age 3 and 8 weeks. At 16 weeks, mothers completed the Babies Need Feeding questionnaire, the Infant Feeding Styles Questionnaire, and the Babies Need Soothing questionnaire. Logistic regression and general linear models examined the effect of study group on infant feeding practices. Moderation analyses explored whether effects varied by feeding mode (any breast milk versus exclusive formula), maternal age (≥ 20 years versus < 20 years), and maternal pre-pregnancy BMI (with obesity versus not).

**Results:**

RP mothers reported more responsive feeding (*p* = 0.005, partial η^2^ = 0.02), lower likelihood of using beverages other than breast milk/formula to soothe their infant (*p* = 0.01, OR = 0.42, 95% CI [0.2–0.8]), and less pressure with cereal than control mothers (*p* = 0.09, partial η^2^ = 0.02). RP mothers also reported less pressure to finish/soothe than controls (*p* = 0.007, partial η^2^ = 0.04); feeding mode (B = 0.74, *p* = 0.003) and maternal age (B = 0.53, *p* = 0.04) moderated this effect. There were no significant group differences in bottle-feeding practices (e.g., adding cereal to bottle, using an appropriate nipple/bottle size), or in context-based or emotion-based food to soothe.

**Conclusions:**

Responsive parenting education influenced some feeding practices of African American mothers. Mothers reported using less pressure, a control-based feeding practice, and more responsive feeding than controls.

**Trial registration:**

Sleep SAAF: A Strong African American Families Study. www.clinicaltrials.gov NCT03505203. Registered 3 April 2018.

**Supplementary Information:**

The online version contains supplementary material available at 10.1186/s12966-022-01366-1.

## Introduction

In the US, 9.5% of infants and toddlers younger than age 2 years are at or above the 95^th^ percentile in weight-for-length [1], increasing the risk of later obesity [2, 3]. The first 1,000 days, or the period from conception to child age 2 years, is recognized as a pivotal developmental period for the prevention of childhood obesity [4]. Interventions using a responsive parenting (RP) framework—defined as parenting that is developmentally appropriate, prompt, and contingent to a child’s needs [5]—have shown promise in improving parent and infant behaviors that contribute to later obesity and a variety of other adaptive outcomes in children [6–8]. Further, guidance from the Robert Wood Johnson Healthy Eating Research expert committee recommends responsive feeding, a component of RP, to foster optimal child development and prevent excessive weight gain [9].

There has been little research related to RP interventions among African American mother-infant dyads, despite greater use of maternal pressure to eat (i.e., attempting to get their child to consume a larger amount) in this population [10], which may contribute to rapid weight gain during infancy [4] and obesity during childhood [11]. To address this gap, the Sleep SAAF (Strong African American Families) study tested the effects of an RP intervention compared to a safety control on rapid infant weight gain among primiparous African American mothers [12]. The RP intervention was adapted from the INSIGHT (Intervention Nurses Start Infants Growing on Healthy Trajectories) RP intervention, a nurse-delivered curriculum that provided primiparous mothers with guidance on infant sleep, feeding, soothing, and interactive play [13]. The RP curriculum included messaging on contingent feeding (i.e., feeding in response to infants’ hunger and satiety cues), using alternatives to food to soothe, and developmentally-appropriate feeding (i.e., delaying the introduction to solids) to promote infants’ development of self-regulation of food intake [14, 15]. INSIGHT methods and intervention materials were tailored for African American families in Sleep SAAF (e.g., emphasis on healthy sleep during recruitment rather than an explicit focus on feeding practices and/or infant weight, implementation by African American community research associates (CRAs), a booster 8-week session, photographs of African American mothers and infants; see [12] and [16]). INSIGHT infants randomized to the RP group had slower weight gain during the first 6 months, reduced overweight at age 1 year [8], and mothers showed improved responsive feeding practices (i.e., less control-based and more structure-based feeding) compared to control mothers [17]. In Sleep SAAF, RP infants were less likely to experience upward crossing of two major weight-for-age percentile lines and there were small, non-significant effects of the RP intervention on conditional weight gain [16].

Building on previous literature showing positive effects of the INSIGHT intervention on responsive feeding practices among a sample of predominantly White families, the primary aim of the current analysis was to assess the effect of the Sleep SAAF RP intervention on African American mothers’ infant feeding practices at infant age 16 weeks. We hypothesized that mothers randomized to the RP group would report greater use of developmentally appropriate bottle-feeding practices, greater use of responsive feeding practices, and less control-based feeding practices compared to control mothers. The exploratory aim was to examine whether study group effects varied by feeding mode, maternal age, and maternal pre-pregnancy BMI. Feeding mode was examined based on the known low breastfeeding rates among African American mothers [18], maternal age was examined based on the differences between INSIGHT (≥ 20 years of age) and Sleep SAAF (≥ 17 years of age) in maternal age eligibility criteria, and maternal pre-pregnancy BMI was examined based on previous findings indicating that mothers with obesity engage in less responsive feeding [19]. Given the exploratory nature of this aim, we did not make a priori hypotheses.

## Subjects and methods

### Participants and study design

Mother-infant dyads were recruited into Sleep SAAF shortly after delivery (infant mean age = 1.5 days at enrollment) from the mother/infant nursery at Augusta University Medical Center (AUMC) in Augusta, GA. Recruitment began in the spring of 2018 and continued through the spring of 2021, with a recruitment pause from March 9, 2020 – August 31, 2020 due to the COVID-19 pandemic. Primiparous mothers ≥ 17 years of age were eligible if they self-identified as African American/Black, had a full-term (≥ 37 weeks gestational age) singleton pregnancy, were English speaking, lived within ≤ 75 miles of Augusta, and had an infant ≥ 2500 g at birth. Dyads were excluded if the mother had a known medical condition that could impact postnatal care (e.g., major mental illness, substance use disorder), if the infant had a medical condition that would impact feeding or growth (e.g., cleft palate), if there was an adoption plan in place, or if there was a plan to move out of the area within four months of delivery.

Consent was obtained by the project’s recruitment coordinator in the hospital. Mother-infant dyads were visited at home at approximately 1, 3, 8, and 16 weeks postpartum by trained community research associates (CRAs) from the Center for Family Research at the University of Georgia. Participants were randomized to either the RP or safety control group after completion of the 1-week data collection visit. The randomization scheme stratified on sex-specific birth weight for gestational age (< 50th percentile or > 50th percentile) and intended feeding mode (breastfeeding or formula). Intervention content was delivered at the 3-week visit (which lasted 90–120 min on average) with booster training at the 8-week visit (which lasted 45–60 min on average). Participating mothers received $50 after the 1-week visit, $50 after the 3-week visit, $100 after the 8-week visits, and $100 after the 16-week visit. Two hundred and twelve mother-infant dyads were randomized. At 16 weeks, 194 (92%) dyads provided data. Sleep SAAF was approved by the Augusta University Institutional Review Board and was registered on www.clinicaltrials.gov (NCT03505203). See Supplemental Fig. 1 for a participant flow chart. Further details on study design and recruitment/eligibility have been published previously [12, 16].

### RP intervention feeding and crying-related components

Intervention content provided messaging on RP in the context of infant feeding, crying, sleeping, and interactive play, drawing from the INSIGHT 2-week RP curriculum [13]. Guidance on infant feeding included teaching mothers to recognize hunger cues (rooting, mouthing, bringing hand to mouth) and fullness cues (letting go of nipple, falling asleep, turning head away, interest in other things). Mothers were also taught that crying is the last sign of hunger and encouraged to keep track of when they last fed their baby and to watch for signs that their baby was hungry if it had been more than a couple of hours since the last feeding. Expectations for typical feeding frequency during the day and night for breastfed and formula fed infants were also discussed. Mothers were given education on age-appropriate bottle sizes, milk/formula volumes, use of slow-flow bottle nipples for infants under 4 months to prevent overfeeding or choking, and how to use fullness cues, rather than the amount of milk in the bottle, to determine when to terminate a feeding. Mothers were also advised that breast milk or formula is best for infants of that age, to delay the introduction of other beverages and solid foods until the infant was 6 months old, and to avoid adding infant cereal to a bottle.

Guidance on crying included information on reasons for infant crying, that crying does not always indicate hunger, how to discriminate hunger from other reasons for infant crying, and how to use alternative soothing strategies rather than feeding. Mothers were also taught the “5 S’s” soothing strategies that they could use to soothe their crying baby: Shushing, Swinging, Side/Stomach Position, Sucking, and Swaddling [20]. Strategies for dealing with night waking to promote infant self-soothing were also highlighted, including allowing the mother a brief time for the infant to self-soothe before intervening to soothe the infant.

### Safety control intervention

Mothers in the control group received a developmentally-appropriate child safety intervention. CRAs taught mothers about sudden infant death syndrome (SIDS) facts and myths, reducing baby’s risk, and the importance of good health care; taking care of a crying baby; finding caretakers for baby; and food safety, including formula and bottle handling, preparation, and storage. Additional information included home and car seat safety. The home visits were matched in length and intensity to the RP intervention and avoided messages related to RP.

### Measures

Maternal age, self-identified race, and infant gestational age were extracted from electronic medical records. Mothers self-reported pre-pregnancy weight at enrollment and their height was measured in duplicate (Seca 274, Hanover, MD) by trained research staff; these measurements were used to calculate pre-pregnancy BMI. Mothers reported family demographics at 1 week postpartum and infant feeding practices at 16 weeks postpartum using Qualtrics, a secure web-based survey tool.

### Infant feeding practices

#### Bottle-feeding practices

Mothers reported developmentally-relevant infant feeding practices using the Babies Need Feeding component from the Baby’s Basic Needs Questionnaire [21]. Mothers were asked about bottle-feeding practices such as adding cereal to the bottle, bottle and nipple size, and number of day and night feedings. Only mothers who reported any formula feeding were asked about their bottle-feeding practices.

####  Introduction to other beverages and solids

Mothers reported whether they had introduced other beverages (i.e., anything other than breast milk or formula) and/or solid foods to their baby using the Babies Need Feeding component from the Baby’s Basic Needs Questionnaire [21].

#### Pressure-based feeding practices

Mothers rated developmentally-relevant items (*n* = 17) from the Infant Feeding Style Questionnaire (IFSQ)[22] that were behavioral targets of the RP intervention. Items assessed mothers’ use of pressure and responsiveness, as well as avoiding the use of food to soothe a fussy infant. Because we utilized a subset of items from the original measure, we conducted an exploratory factor analysis to create subscales. A principal components extraction with an oblimin rotation was used. The scree plot indicated that 2 or 3 factors would be most appropriate. Both were examined and items with factor loadings < 0.45 were iteratively removed. Restricting to 3 factors gave factor loadings with simple structure: pressure to finish/soothe (7 items; e.g., “The best way to make an infant stop crying is to feed him or her”), pressure with cereal (3 items; e.g., “Putting cereal in a bottle is good because it helps an infant feel full”), and responsiveness to infant cues (3 items; e.g., “I let my baby decide how much to eat”). The inter-factor correlation was moderate between pressure-based factors (*r* = 0.32, *p* < 0.001) but the responsiveness factor was not significantly correlated with either pressure-based factor (pressure to finish/soothe *r* = 0.04, *p* = 0.66, pressure with cereal *r* = -0.01, *p* = 0.89). Cronbach alphas were acceptable for pressure to finish/soothe (α = 0.76) and pressure with cereal (α = 0.76), but not responsiveness (α = 0.41). Therefore, the 3 responsiveness items were analyzed individually to explore intervention effects on these behaviors. Supplemental Table 1 includes all items and factor loadings.

#### Food to soothe

Mothers reported whether they used breast milk/formula, other beverages, or solid foods as a method to soothe their infant, and the use of food to soothe infant fussiness not related to hunger using the Babies Need Soothing items (*n* = 16) from the Baby’s Basic Needs Questionnaire [17, 21]. An exploratory factor analysis was conducted to create food to soothe subscales. A principal components extraction with an oblimin rotation was used. The scree plot indicated 3 factors would be most appropriate and items with factor loadings < 0.45 were iteratively removed. Restricting to 3 factors gave factor loadings with simple structure: context-based (6 items; e.g., “How likely are you to use food to calm your baby in church or other place of worship?”), emotion-based (5 items; e.g., “How likely are you to use food to calm your baby when you are stressed?”), and sleep-based (2 items; e.g., “How likely are you to use food to calm your baby when your baby wakes during the night?”) food to soothe. The inter-factor correlations were moderate (emotion-based and context-based food to soothe *r* = 0.35, *p* < 0.001; emotion-based and sleep-based food to soothe *r* = 0.31, *p* = 0.005; context-based and sleep-based food to soothe *r* = 0.48, *p* < 0.001). Cronbach alphas were good for context-based (α = 0.87), emotion-based (α = 0.87), and sleep-based (α = 0.72) food to soothe. Supplemental Table 2 includes all items and factor loadings.

### Statistical analyses and power

Data were analyzed using SAS 9.4 (SAS Institute, Cary, NC). The effect of study group on infant feeding practices was examined using logistic regression for categorical outcomes and general linear models for continuous outcomes. A dummy variable was created to assess whether infants were seen within the designated window for the 16-week visit (i.e., 15–18 weeks); this variable was tested as a covariate and retained if significant. Exploratory analyses were conducted to examine the moderating effect of feeding mode (any breast milk versus exclusive formula at 16 weeks), maternal age category (≥ 20 years versus < 20 years to match eligibility criteria from INSIGHT [13]), and maternal pre-pregnancy BMI (with obesity defined as BMI ≥ 30 versus not) on the use of pressure-based and food to soothe subscales. Mothers who responded that they were ‘never’ the person to feed their baby (n = 3) or did not answer this question (n = 1) were removed from the current analysis given our focus on feeding, resulting in a final analytic sample of 190 mother-infant dyads. Statistical significance was determined a priori as *p* < 0.05 (two-tailed).

Statistical power for the study was originally calculated for the primary outcome of between-group differences in conditional weight gain scores from 3 to 16 weeks, which is reported elsewhere [16]. Target enrollment was 300 mother-infant dyads based on the INSIGHT effect size of approximately 0.4 for infant conditional weight gain at 6 months [8]. G Power 3.1.9.2 [23] was used to estimate analytic power for the current sample of 190 dyads to identify RP intervention effects on maternal feeding practices; 80% power could be achieved with an effect size of *d* = 0.41 (at 5%, two-tailed Type 1 error rate).

## Results

Table 1 presents family demographics. Primiparous mothers were African American/Black (100%), non-Hispanic (99%), in a romantic relationship (61.1%), had some high school or completed high school (60%), and participated in SNAP (47.3%) and WIC (76.1%). Slightly more than half (53.7%) reported any breastfeeding at the initial 3-week intervention visit. These demographic variables did not differ by study group.

**Table 1:** Participant demographics by study group

	**RP (*n* = 96)**	**Control (*n* = 94)**
**Infant**		
Male sex, n (%)	47 (49.0)	44 (46.8)
Gestational age (weeks), mean (SD)	39.1 (1.03)	39.1 (1.08)
Birth weight (kg), mean (SD)	3.0 (0.38)	2.99 (0.36)
Birth length (cm), mean (SD)	48.73 (1.76)	48.68 (1.59)
**Mother**		
Age (years), mean (SD)	23.65 (4.98)	22.16 (3.99)
Age 20 years or older, n (%)	86 (79.6)	72 (67.9)
Any breast feeding at 16wk, n (%)	22 (22.9)	21 (21.4)
Pre-pregnancy BMI, mean (SD)	27.71 (8.66)	28.49 (8.06)
Obese pre-pregnancy BMI, n (%)	37 (34.6)	33 (31.7)
Gestational weight gain (kg), mean (SD)	16.41 (9.26)	13.94 (8.34)
Diabetes during pregnancy, n (%)	6 (6.25%)	7 (7.4%)
Romantic status, n (%)		
Single	35 (36.5%)	39 (41.5%)
Married / living together	15 (15.63%)	7 (7.5%)
Married, but not living together	0 (0.0%)	0 (0.0%)
Living together	29 (30.2%)	30 (31.9%)
Involved/steady/not living together	16 (16.7%)	18 (19.1%)
Involved/on–off again relationship	1 (1.0%)	0 (0.0%)
Education, n (%)		
Some high school (9–11)	12 (12.5%)	10 (10.6%)
High school graduate	45 (46.9%)	47 (50.0%)
Some college or technical school	24 (25.0%)	26 (27.7%)
Completed college	9 (9.4%)	9 (9.6%)
Post graduate training degree	6 (6.3%)	2 (2.1%)
Federal Nutrition Assistance, n (%)		
SNAP participation (yes)	47 (50.5%)	40 (44.0%)
WIC participation (yes)	75 (79.8%)	68 (72.3%)

### Study group effects on bottle-feeding at 16 weeks

Table 2 presents results for all infant feeding practices by study group. There were no significant study group differences in mothers’ bottle-feeding practices. Mothers from both groups reported adding cereal to their infant’s bottle (RP 45.1%, control 55.2%), using an age-appropriate slow-flow nipple (RP 57.14%, control 47.5%) and using an age-appropriate bottle < 8 oz (RP 73.4%, control 73.8%) at 16 weeks. Number of day feedings (RP mean = 6.05, control mean = 6.09) and night feedings (RP mean = 4.00, control mean = 4.34) at 16 weeks also did not differ significantly by study group.

**Table 2:** Effect of RP intervention on maternal feeding practices

	**RP**	**Control**	***p***	**Odds ratio [95% CI] or Partial η**^**2**^
**Bottle-feeding practices**				
Currently adding cereal to bottle^a^				
	37/82 (45.1%)	48/87 (55.2%)	0.28	0.71 [0.4-1.3]
Uses slow flow nipple^a^				
	44/77 (57.14%)	38/80 (47.5%)	0.27	1.43 [0.8-2.7]
Uses bottle < 8 oz^a^				
	58/79 (73.4%)	62/84 (73.8%)	0.79	0.91 [0.4-1.9]
Daytime (7am-7pm) feedings – mean (SD)^a^				
	6.05 (1.94) *n* = 96	6.09 (2.07) *n* = 95	0.74	0.0006
Nighttime (7 pm-7 am) feedings – mean (SD)				
	4.00 (1.77) *n* = 96	4.34 (2.13) *n* = 95	0.24	0.007
**Introduction to other beverages/solids**				
Introduced other beverages^a^				
	26/96 (27.1%)	35/94 (37.2%)	0.23	0.67 [0.4-1.3]
Introduced solid foods^a^				
	18/96 (18.8%)	23/94 (24.5%)	0.74	0.86 [0.4-2.0]
**Pressure-based and responsive feeding**				
Pressure to finish/soothe factor – mean (SD)				
	2.04 (0.72), *n* = 96	2.33 (0.75), *n *= 94	0.007	0.04
Pressure with cereal factor – mean (SD)				
	2.70 (1.22), *n* = 96	2.99 (1.18), *n* = 94	0.09	0.02
I let my baby decide how much to eat				
	4.00 (1.25), *n* = 95	3.40 (1.59), *n* = 94	0.005	0.04
My baby lets me know when s/he is full				
	4.63 (0.73), *n* = 96	4.63 (0.66), *n* = 94	0.98	0.000
My baby lets me know when s/he is hungry				
	4.69 (0.58), *n* = 95	4.60 (0.71), *n* = 93	0.33	0.005
**Food to soothe**				
Use of breast milk/formula to soothe				
	87/95 (91.6%)	91/99 (91.9%)	0.80	0.88 [0.3-2.5]
Use of other beverages to soothe				
	16/96 (16.7%)	32/97 (33.0%)	0.01	0.42 [0.2-0.8]
Use of solid foods to soothe^a^				
	21/96 (21.9%)	35/97 (36.1%)	0.08	0.53 [.3-1.1]
Context-based food to soothe – mean (SD)				
	2.88 (1.01), *n* = 94	3.01 (0.96), *n* = 92	0.37	0.004
Emotion-based food to soothe – mean (SD)				
	2.10 (1.08), *n* = 92	2.00 (1.04), *n* = 88	0.99	0.0000
Sleep-based food to soothe – mean (SD)				
	3.79 (1.04), *n* = 93	3.82 (1.00), *n* = 91	0.82	0.0003

^a^ Indicates models in which the study window timing for the 16-week visit was significant and therefore retained

### Study group effects on introduction of other beverages and solids at 16 weeks

There were no significant study group differences in mothers’ introduction of other beverages and solid foods. A minority of mothers in both groups reported introducing other beverages in their infant’s bottle (RP 27.1%, control 37.2%) and introducing solid foods (RP 18.8%, control 24.5%) at 16 weeks.

### Study group effects on pressure-based and responsive feeding practices at 16 weeks

Mothers in the RP group used significantly less pressure to finish/soothe than mothers in the control group (RP *M* = 2.04, control *M* = 2.33, *p* = 0.007). Mothers in the RP group also reported less pressure with cereal, though not statistically significant (RP *M* = 2.70, control *M* = 2.99, *p* = 0.09). Mothers in the RP group reported significantly higher endorsement of the statement “I let my baby decide how much to eat” than mothers in the control group (RP *M* = 4.00, control *M* = 3.40, *p* = 0.005). There were no significant study group differences in mothers’ responses to the two other responsive feeding questions (“My baby lets me know when s/he is full” and “My baby lets me know when s/he is hungry”).

### Study group effects on food to soothe at 16 weeks

Most mothers reported using breast milk/formula to soothe their infant (RP 91.6% RP, control 91.9%), with no difference between study groups. Mothers in the RP group were significantly less likely than mothers in the control group to report using other beverages to soothe their infant (RP 16.7%, control 33.0%; *p* = 0.01). Mothers in the RP group were also less likely to report using solid foods to soothe their infant, though not statistically significant (RP 21.9%, control 36.1%; *p* = 0.08). There were no study group differences in mothers’ use of context-based, emotion-based, or sleep-based food to soothe.

### Exploratory analyses testing moderation by maternal characteristics

#### Feeding mode

**Fig. 1:**
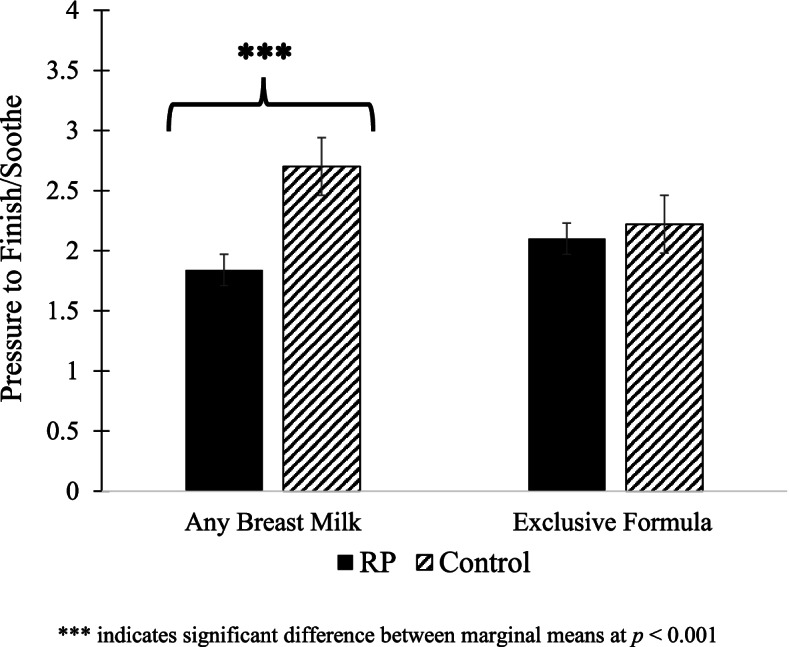
Feeding mode moderates study group effects on mothers’ use of pressure to finish/soothe

At 16 weeks, 22.9% of RP mothers and 21.4% of control mothers reported any breast milk feeding and 77.1% of RP mothers and 78.6% of control mothers reported exclusive formula feeding; these rates did not differ by study group. Feeding mode moderated the study group effect on the use of pressure to finish/soothe (B = 0.74, *p* = 0.003), such that compared to the control group, the RP intervention resulted in lower use of pressure to finish/soothe only among mothers who reported feeding any breast milk (*p* = 0.0001), but not among mothers who exclusively formula fed (*p* = 0.31; Fig. 1). There were no significant interactions between feeding mode and study group on mothers’ use of pressure with cereal, context-based food to soothe, or emotion-based food to soothe (data not shown).^1^

#### Maternal age

**Fig. 2:**
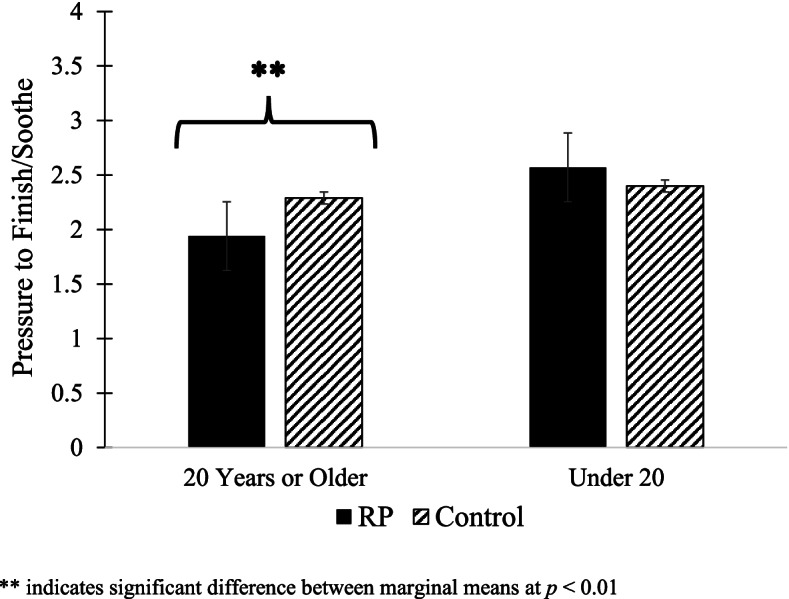
Maternal age moderates study group effects on mothers’ use of pressure to finish/soothe

**Fig. 3:**
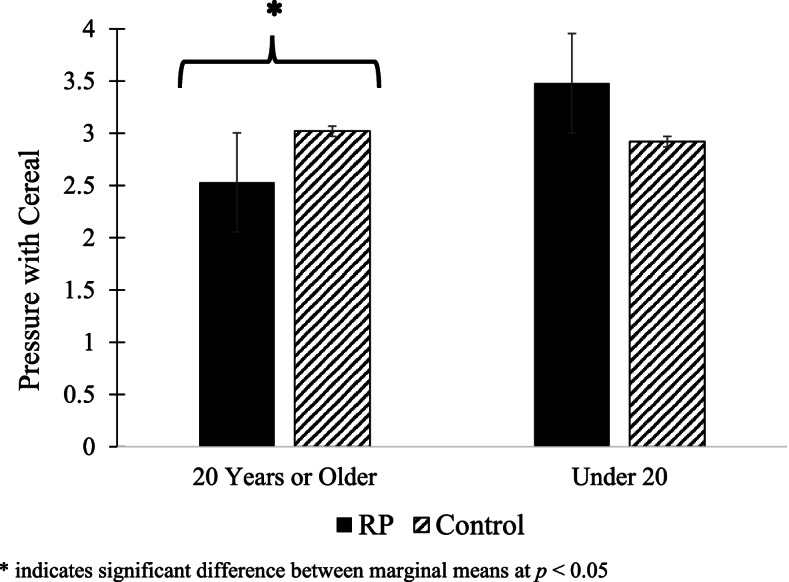
Maternal age moderates study group effects on mother’s use of pressure with cereal

At 16 weeks, 79.6% of RP mothers and 67.9% of control mothers were age 20 years or older and 20.4% of RP mothers and 32.1% of control mothers were younger than age 20 years; these rates were significantly different between groups (*p* = 0.05). Maternal age moderated the study group effects on the use of pressure to finish/soothe (B = 0.53, *p* = 0.04) and pressure with cereal (B = 1.04, *p* = 0.01). Relative to the control group, the RP intervention resulted in less pressure to finish/soothe (*p* = 0.003) and pressure with cereal (*p* = 0.02) among older, but not younger, mothers (Figs. 2 and 3). There were no significant interactions between maternal age and study group on mothers’ context-based or emotion-based food to soothe (data not shown).

#### Maternal pre-pregnancy BMI

At pre-pregnancy, 34.6% of RP mothers and 31.7% of control mothers met the criteria for obesity and 65.4% of RP mothers and 68.3% of control mothers did not; these rates did not differ by study group. There were no significant interactions between maternal pre-pregnancy BMI and study group on any feeding practice (data not shown).

## Discussion

Consistent with hypotheses, the Sleep SAAF RP intervention designed for primiparous African American mother-infant dyads improved some infant feeding practices. Mothers randomized to the RP intervention reported less pressure-based feeding, a control-based feeding practice, and more responsive feeding compared to control mothers. Additionally, although RP and control mothers did not differ in their use of breastmilk/formula to soothe their infants, RP mothers were significantly less likely to use other beverages to soothe their infants. There were no study group differences in the use of food to soothe or bottle-feeding practices (e.g., adding cereal to the bottle, bottle and nipple size, number of feedings, introduction of other beverages or solid foods). Exploratory analyses revealed that feeding mode and maternal age moderated RP intervention effects on mothers’ use of pressure-based feeding with greater effects observed among mothers who used some breast feeding and among older mothers. Together, these findings suggest that the Sleep SAAF RP curriculum influenced some aspects of how African American mothers fed their firstborn infants.

RP mothers reported using less pressure to finish/soothe, and marginally less pressure with cereal, compared to control mothers, which is consistent with previous research. For example, in the INSIGHT study which included nurse home visits at ages 3, 16, 28, and 40 weeks, RP intervention effects on pressure-based feeding were identified as early as 8 and 16 weeks [17]. In the Mothers & Others trial among African American families, which included prenatal home visits at 30 and 34 weeks and postnatal visits at infant ages 3, 6, 9, and 12 months, RP effects on African American mothers’ pressure-based feeding were identified at infant age 15 months, but not at baseline or 3 months [24]. In Sleep SAAF, RP mothers also reported using more responsive feeding (i.e., “I let my baby decide how much to eat”) compared to control mothers. Given the known associations between pressure-based feeding and rapid infant weight gain [21, 25, 26], as well as protective effects of responsive feeding [14, 15], reducing pressure-based feeding and promoting responsive feeding are important early intervention targets to promote healthy growth and development during infancy.

RP mothers were less likely to use other beverages to soothe their infant, and marginally less likely to use solid foods to soothe their infant, compared to control mothers. The Sleep SAAF RP curriculum included messaging on identifying reasons for infant crying as well as alternative soothing strategies such as shushing and swaddling [20], which may help explain these findings. However, no study group differences were observed in mothers’ use of context-based, emotion-based, or sleep-based food to soothe. Nearly all mothers (92%) from both the RP and control groups reported using breast milk/formula to soothe, which may help explain the null findings. RP interventions conducted in predominantly White families have demonstrated intervention effects on mothers’ use of food to soothe as early as age 8 weeks [17]. Greater attention to other factors affecting African American mothers’ infant feeding practices, including broad social determinants of health (e.g., access to prenatal healthcare, adequate paid maternity leave; [24]) as well as sociocultural beliefs [28, 29], may strengthen RP intervention messaging and reduce use of breastmilk or formula to soothe a distressed infant.

In the current study, about half of the mothers in each group reported adding cereal to the bottle, and approximately a quarter from each group reported introducing solid foods by 16 weeks. Both of these practices are associated with rapid infant weight gain [30–32] yet are common among African American families [28, 33–35]. Further, the lack of intervention effects on mothers’ bottle-feeding practices is surprising given that the RP curriculum included messaging on age-appropriate bottle and nipple sizes, avoiding adding infant cereal to the bottle, and delaying the introduction of other beverages and solid foods until age 6 months. Previous research has identified that early introduction of solids as well as adding cereal to the bottle early in an infant’s life are considered cultural norms for African American mothers [28], but it is not yet well understood why this is the case. In qualitative studies, African American mothers have reported adding cereal to their infant’s bottle to help them sleep through the night [29, 36]. Previous work has also highlighted the prominence of intergenerational influences on African American mothers’ bottle-feeding practices [28, 36], suggesting that these behaviors may be entrenched and difficult to change. Other research has indicated that low-income mothers often receive messages from their social networks that conflict with infant feeding recommendations [37] and that low-income mothers may perceive that infant feeding recommendations do not fit with their family’s needs [38]. Given that the current intervention did not change these practices (but did in INSIGHT; [17]), additional research is needed to identify more culturally-specific strategies to change these practices for African American mothers. At the same time, we note that INSIGHT RP intervention effects were not observed for bottle-feeding practices until age 20 weeks [17], raising the possibility that differences between the RP and control interventions might have been observed had measurement timepoints occurred beyond infant age 16 weeks.

Feeding mode moderated intervention effects on mothers’ pressure-based feeding, such that RP mothers reported lower pressure to finish/soothe compared to controls for mothers who reported any breast milk feeding, but not for mothers who reported exclusive formula feeding. Previous research shows that feeding from the breast promotes mother-infant bonding [39], which is a foundational concept in RP. Further, some research suggests that mothers are more sensitive to their infant’s hunger and fullness cues (a responsive feeding practice) when feeding from the breast compared to the bottle [40], which may partially explain differences in efficacy by feeding mode. This hypothesis is tentative, however, given that we did not assess whether mothers were feeding breast milk from the breast or bottle. Maternal age also moderated intervention effects on mothers’ pressure-based feeding, such that RP mothers reported less pressure to finish/soothe and pressure with cereal compared to controls only for mothers aged 20 years or older, but not for mothers younger than age 20 years. Previous research suggests that older mothers engage in more general RP practices compared to younger mothers [41–44], which may partially explain differences in efficacy by maternal age. Maternal pre-pregnancy BMI was not a significant moderator of RP intervention effects (and was not significantly associated with mothers’ feeding practices), which is inconsistent with previous findings that mothers with obesity engage in less responsive feeding [19]; these patterns warrant further investigation. Given findings in the current study, and the existing RP literature, future studies should include additional messaging to help reduce pressure-based feeding among exclusive formula feeding mothers as well as younger mothers.

Some limitations of the current study include the use of maternal self-report to assess infant feeding practices, which may be subject to social desirability bias. Future research should examine effects of RP intervention on observed infant feeding practices. The sample from the current study was also limited to primiparous African American mothers from the southeastern US, which limits the generalizability of these findings. In addition, due to the onset of the COVID-19 pandemic, the sample was smaller than intended (*n* = 300), which may have limited our ability to detect intervention effects. Additional research with a larger sample is needed to better understand infant feeding practices among African American mothers throughout the US. Finally, it is important to acknowledge that the Sleep SAAF RP intervention was adapted from INSIGHT, an existing intervention that was shown to be effective among a predominantly White sample of mothers. Although the Sleep SAAF RP program was tailored in some ways for African American families, an alternate approach in which the intervention was developed solely to meet the specific needs of African American mothers may yield different outcomes and should be considered in future work with this population.

## Conclusions

In summary, the Sleep SAAF RP intervention improved some infant feeding practices among African American mothers. Relative to controls, mothers in the RP intervention reported lower use of pressure-based feeding, higher use of responsive feeding, and were less likely to use beverages other than breast milk/formula or solids to soothe their infants. Findings suggest that African American mothers’ early infant feeding practices are modifiable with RP intervention and support the viability of these types of interventions to enhance responsive feeding among African American families.

## Supplementary Information

**Additional file 1: Supplemental Figure 1.** Study CONSORT diagram.

**Additional file 2: Supplemental Table 1.** Factor loadings for selected IFSQ questionnaire items.

**Additional file 3:** **Supplemental Table 2. **Factor loadings for selected Babies Need Soothing (BNS) questionnaire items.

## Data Availability

Data described in the manuscript, code book, and analytic code will be made available upon request from the corresponding author.

## Abbreviations

RP: Responsive Parenting
INSIGHT: Intervention Nurses Start Infants Growing on Healthy Trajectories
SAAF: Strong African American Families
CRA: Community Research Associate
SIDS: Sudden Infant Death Syndrome
IFSQ: Infant Feeding Styles Questionnaire
SNAP: Supplemental Nutrition Assistance Program
WIC: Special Supplemental Nutrition Program for Women, Infants, and Children

## Footnotes

1 There were no significant main effects of feeding mode, maternal age, or maternal pre-pregnancy BMI on mothers’ pressure to finish/soothe, mothers’ pressure with cereal, context-based food to soothe, or emotion-based food to soothe.
