# Supplementary material for: Sleep SAAF responsive parenting intervention improves mothers’ feeding practices: a randomized controlled trial among African American mother-infant dyads
Source: Int J Behav Nutr Phys Act. 2022 Oct 1;19:129. doi: 10.1186/s12966-022-01366-1 (PMC9526457; doi:10.1186/s12966-022-01366-1)
Supplement: Supplementary file 1 — Additional file 1: Supplemental Figure 1. Study CONSORT diagram. [file 12966_2022_1366_MOESM1_ESM.docx]

**Supplemental Figure 1**

Study CONSORT diagram


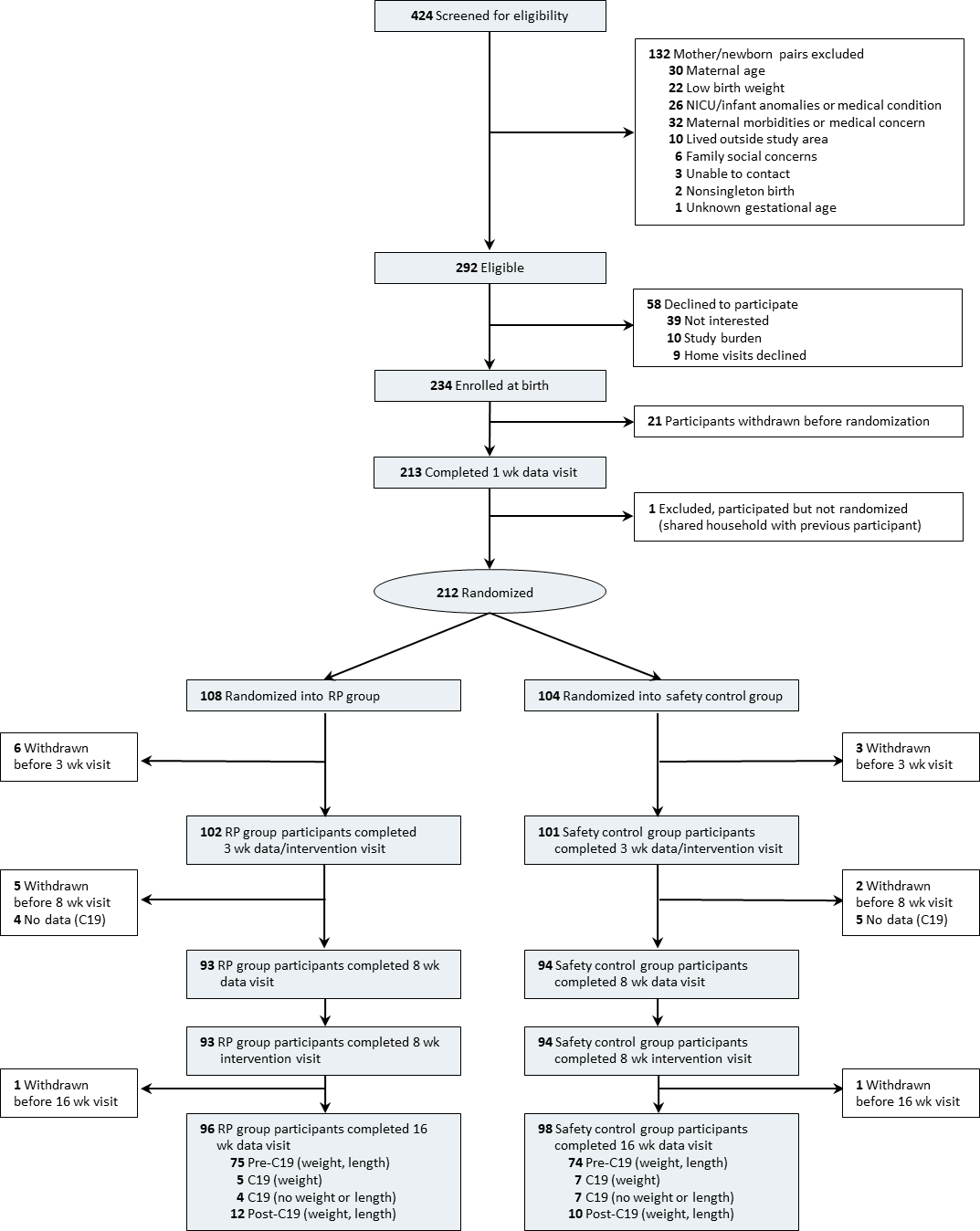


*Notes*. The number screened for eligibility only includes primiparous African American mothers; mothers who were another race or multiparous were not recorded. Mother/newborn pairs meeting more than one exclusion criteria are listed only once in the exclusion breakdown. RP = Responsive Parenting. C19 = COVID-19 pandemic.
