# Supplementary material for: Sleep SAAF responsive parenting intervention improves mothers’ feeding practices: a randomized controlled trial among African American mother-infant dyads
Source: Int J Behav Nutr Phys Act. 2022 Oct 1;19:129. doi: 10.1186/s12966-022-01366-1 (PMC9526457; doi:10.1186/s12966-022-01366-1)
Supplement: Supplementary file 2 — Additional file 2: Supplemental Table 1. Factor loadings for selected IFSQ questionnaire items. [file 12966_2022_1366_MOESM2_ESM.docx]

**Supplemental Table 1**

Factor loadings for selected IFSQ questionnaire items

| **IFSQ question text** | **Factor 1: Pressure finishing/soothing** | **Factor 2: Pressure cereal** | **Factor 3: Responsiveness** |
| --- | --- | --- | --- |
| When an infant cries it usually means he or she needs to be fed.^2^ | **.59^1^** | -.08 | -.09 |
| I feed my baby to keep her/him from crying.^3^ | **.59^1^** | .06 | .08 |
| When my baby cries, I immediately feed him/her.^3^ | .**67^1^** | -.02 | .18 |
| I try to get my baby to finish his/her breastmilk or formula.^3^ | **.51^1^** | -.01 | -.10 |
| The best way to make an infant stop crying is to feed him or her.^2^ | **.72^1^** | .16 | .11 |
| I try to get my baby to eat even if s/he seems not hungry.^3^ | **.65^1^** | .01 | .05 |
| It’s important that an infant finish all of the milk in his or her bottle.^2^ | **.64^1^** | -.15 | -.09 |
| Cereal in the bottle will help an infant sleep through the night.^2^ | -.10 | **.91^1^** | 0 |
| Putting cereal in a bottle is good because it helps an infant feel full.^2^ | -.15 | **.93^1^** | -.03 |
| An infant less than 6 months old needs more than formula or breastmilk to be full.^2^ | .14 | **.49^1^** | -.34 |
| I let my baby decide how much to eat.^3^ | -.01 | -.01 | **.64^1^** |
| My baby lets me know when s/he is full.^3^ | .05 | .16 | **.60^1^** |
| My baby lets me know when s/he is hungry.^3^ | .09 | -.04 | **.63^1^** |
| **Items that did not load** |  |  |  |
| I watch TV while I feed my baby.^3^ | .06 | .13 | .20 |
| I think it is ok to prop an infant’s bottle.^2^ | .07 | .12 | .10 |
| I allow my baby to eat when s/he is hungry.^3^ | -.02 | -.03 | .14 |
| It’s important for the parent to decide how much an infant should eat.^2^ | .12 | .11 | -.27 |

1Factor loadings that contribute to defining each factor.
2Responses are scored on a 5-point Likert scale: 1 = *never* to 5 = *always.*

^3^Responses are scored on a 5-point Likert scale: 1 = *disagree* to 5 = *agree*.
