# Supplementary material for: Sleep SAAF responsive parenting intervention improves mothers’ feeding practices: a randomized controlled trial among African American mother-infant dyads
Source: Int J Behav Nutr Phys Act. 2022 Oct 1;19:129. doi: 10.1186/s12966-022-01366-1 (PMC9526457; doi:10.1186/s12966-022-01366-1)
Supplement: Supplementary file 3 — Additional file 3: Supplemental Table 2. Factor loadings for selected Babies Need Soothing (BNS) questionnaire items. [file 12966_2022_1366_MOESM3_ESM.docx]

**Supplemental Table 2**

Factor loadings for selected Babies Need Soothing (BNS) questionnaire items

| **BNS question text** | **Factor 1: Context-based FTS** | **Factor 2: Emotion-based FTS** | **Factor 3: Sleep-based FTS** |
| --- | --- | --- | --- |
| In a doctor’s waiting room**^2^** | **.72^1^** | .02 | .14 |
| When you are shopping in a store | **.94^1^** | -.07 | -.11 |
| In church or other place of worship | **.84^1^** | .01 | -.08 |
| In the car | **.68^1^** | -.01 | .19 |
| When you are preparing meals | **52^1^** | .12 | .21 |
| When you are on the phone | **.45^1^** | .31 | .20 |
| You are stressed | .20 | **.78^1^** | -.12 |
| You are frustrated or upset | .00 | **.97^1^** | -.16 |
| You are tired | -.07 | **.85^1^** | .06 |
| You are busy | -.15 | **.91^1^** | .04 |
| When nothing else works to calm or distract my baby | .03 | **.46^1^** | .10 |
| When your baby wakes during the night | .00 | -.03 | **.85^1^** |
| Before naptime or bedtime | .09 | .02 | **.65^1^** |
| **Items that did not load or discriminate** |  |  |  |
| To occupy your baby until mealtime | .44 | .22 | .25 |
| When you are getting ready to leave the house | .34 | .70 | .71 |
| While doing household chores | .64 | .64 | .55 |

1Factor loadings that contribute to defining each factor.
2Responses are scored on a 5-point Likert scale: 1 = *never* to 5 = *always.*
